# Reconstruction of the Ascending Reticular Activating System with Diffusion Tensor Tractography in Patients with a Disorder of Consciousness after Traumatic Brain Injury

**DOI:** 10.7759/cureus.1723

**Published:** 2017-09-28

**Authors:** Edgar Gerardo Ordóñez-Rubiano, Jason Johnson, Cesar O Enciso-Olivera, Jorge H Marín-Muñoz, William Cortes-Lozano, Pablo E Baquero-Herrera, Edgar G Ordóñez-Mora, Hernando A Cifuentes-Lobelo

**Affiliations:** 1 Neurosurgery Department, Fundación Universitaria De Ciencias De La Salud, Hospital de San Jose/Hospital Infantil Universitario de San José; 2 Neuroradiology, MD Anderson; 3 Critical Care and Intensive Care Unit. Hospital Infantil Universitario De San José. Bogotá, Colombia, Hospital Infantil Universitario de San José - Fundación Universitaria de Ciencias de la Salud; 4 Neuroradiology. Hospital Infantil Universitario De San José. Bogotá, Colombia., Hospital Infantil Universitario de San José - Fundación Universitaria de Ciencias de la Salud; 5 Neurosurgery, Fundación Universitaria De Ciencias De La Salud, Hospital de San Jose/Hospital Infantil Universitario de San José; 6 Neurosurgery Department. Hospital Infantil Universitario De San José. Bogotá, Colombia, Hospital Infantil Universitario de San José - Fundación Universitaria de Ciencias de la Salud; 7 Neurosurgery Department. Hospital Infantil Universitario De San José. Bogotá, Colombia., Hospital Infantil Universitario de San José - Fundación Universitaria de Ciencias de la Salud

**Keywords:** diffusion tensor imaging, arousal, consciousness, ascending reticular activating system, tractography, traumatic brain injury, neurosurgery

## Abstract

This work describes the reconstruction of the ascending reticular activating system (ARAS) with diffusion tensor tractography in three patients with altered consciousness after traumatic brain injury. A diffusion tensor tractography was performed in three patients with impaired consciousness after a severe traumatic brain injury. A 1.5 T scanner was used to obtain the tensor sequences; axial tensors were acquired. Post-processing was performed, and the mean fractional anisotropy (FA) values were recorded. The FA maps were used to do a manual tracing of the following regions of interest (ROIs): the ventromedial midbrain, the anterior thalamus, and the hypothalamus. Case 1 presented destruction of the right dorsal and ventral tegmental tracts as well as destruction of the right middle forebrain bundle, case 2 had destruction of the right dorsal tegmental tract, and case 3 had destruction of the bilateral ventral and dorsal tegmental tracts, as well as destruction of the right middle forebrain bundle. The affected fibers of the ascending reticular activating system with diffuse axonal injury and the FA values abnormalities in the ascending reticular activating system in three patients with a disorder of consciousness (DOC) after traumatic brain injury are described.

## Introduction

The arousal component of the consciousness is generated by the ascending reticular activating system (ARAS) [1]. It has been largely demonstrated that the ARAS is composed of groups of neurons that project from different nuclei in the brainstem forming a diffuse and complex network that connect to the cortex through thalamic and extra-thalamic pathways. The ARAS integrates its reticular core and its nuclei in the brainstem, as well as its rostral projections directed to the hypothalamus, the thalamus, the basal forebrain, and the cortex [1]. After the development of diffusion tensor imaging (DTI) and fiber tracking post-processing, the neuroanatomic connectivity of the ARAS and its relationship with altered consciousness disorders were carefully described [1]. Description of the ARAS in healthy subjects and in post-mortem formalin brains [1-2] have been reported, as well as some cases with diffuse axonal injury (DAI) after traumatic brain injury (TBI) [3-4]. Analysis of fractional anisotropy (FA) values of white matter tracts in patients after anoxic brain injury [5] and patients after TBI [4] have been performed and a DTI prediction system for long-term neurological outcome in comatose patients has also been reported [6]. The objective of this manuscript is to describe the reconstruction of diffusion tensor tractography (DTT) of the ARAS pathways in three patients with impaired consciousness after TBI.

## Case presentation

Patients and definitions

Clinical and neuroimaging data of three Hispanic male patients with severe TBI, with a disorder of consciousness (DOC) following trauma, admitted at the intensive care unit (ICU) of the Hospital Infantil Universitario de San José, Bogota, Colombia, between January 2015 and January 2017 are presented. The three patients persisted with a DOC after the acute phase of the trauma and were selected for DTI and DTT analysis of the ARAS. Magnetic resonance imaging (MRI) protocols were performed when a hemodynamic stability was achieved and when an electrolytic or any other disturbance that could affect the level of consciousness were ruled out. The MRI was performed on the patients 12, 8, and 11 days, respectively, after the trauma. A multidisciplinary team approach was adopted, which included neurologists, intensivists, and neurosurgeons. During the hospital stay, the clinical assessment included physiotherapy, occupational therapy, speech and language therapy, nursing, and rehabilitation medicine. Clinical decisions always included the family of the patients, who were often present over prolonged periods, even during the ICU stay. A standard clinical evaluation for the diagnostic assessment process included a detailed clinical history; a review of medication; the standard imaging; a standard electroencephalogram when subclinical seizure activity was suspected; and a detailed neurological evaluation, including evaluation with the Glasgow Coma Scale (GCS) in the acute setting and posteriorly with the Disorders of Consciousness Scale (DOCS), a valid diagnostic tool for vegetative state (VS) and minimally conscious state (MCS), consisting of 34 stimuli tests organized in eight subgroups: social knowledge, taste and swallowing, olfactory, proprioceptive and vestibular, auditory, visual, tactile, and testing readiness [7]. All patients were carefully evaluated and assessed every day during their hospital stay by different physicians (intensivists, neurologists, and neurosurgeons).

This work is based on the general principles of human research ethics set forth in the Helsinki Declaration. The purpose of the study is to obtain scientific knowledge for a better diagnosis and a more precise evaluation that may eventually help to predict or improve the neurological outcome of these patients. It adopts the resolution 8430 of 1993 of the Ministry of Health of Colombia and is based on the definitions of risk contained in the corresponding article of that law, which classifies this study as a risk-free research, since it is a retrospective study based on medical records information. Authorization was requested to our institutional ethics committee to include the information of the patients in this study, preserving their identity both in the analysis of the information and in the images presented. This is a retrospectively reviewed case series with approval by the Fundación Universitaria de Ciencias de la Salud, Bogota, Colombia Institutional Review Board. For an adequate interpretation of the clinical status of the patients, for all the terms mentioned in this manuscript regarding any DOC, the corresponding definitions were taken from the clinical guidelines of the Royal College of Physicians [8], including the definitions of consciousness, awareness, wakefulness, coma, VS, MCS, etc.

Imaging acquisition

A General Electric Signa Excite HDXT (1.5-T GE Healthcare, Milwaukee, WI, USA) was used to obtain the images. From each patient, the following sequences were acquired: 1) axial T1-weighted structural/anatomical; 2) axial T2-weighted; 3) axial diffusion weighted imaging (DWI), and 4) axial DTI. Each structural image in T1 has 140 slices, 1 mm thick, without GAP (free space), matrix = 320 x 192, repetition time (TR) = 650 ms, echo time (TE) = 22 ms, field of view (FOV) = 22, and acquisition time = 2 min and 35 seconds, covering the entire brain volume. The structural images in T2 contain 22 cuts (6 mm thickness, GAP 1 mm (free space), matrix = 320 x 256, TR = 6.000 ms, TE = 97.44 ms, FOV = 24), and acquisition time = 1 min and 20s, covering the entire brain volume. Each DWI image has 30 slices (2 mm thickness, without free space, resolution = 2 mm isotropic, matrix = 128 x 128, TR = 1000, TE = 102.3ms, flip angle = 90), planar imaging and acquisition time = 6 min, covering the entire brain volume. For the isometric DTI sequence, a spin echo-planar (EPI) sequence with 24 directions were used in an axial plane without angulation. Images were obtained from the base from the skull to the vertex. Each axial tensor sequence has 920 images, matrix = 100 x 100, TR = 14000 - 17000, TE = minimum, thickness 2.5, spacing = 0.0, NEX = 1, Pixel = 2.5, FOV = 250, b value = 1000, acquisition time = 7 min.

Processing

**Figure 1:**
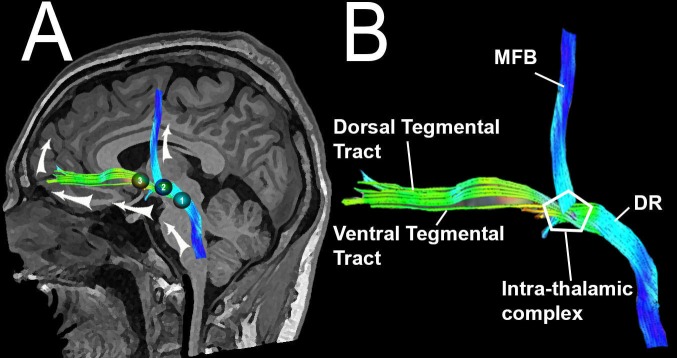
Schematic illustration of the reconstruction of the ascending reticular activating system (ARAS) fiber tracts with tractography in a normal subject. (A) The three regions of interest (ROIs) are represented with numbers: 1) midbrain, 2) thalamus, 3) hypothalamus. (B) The different tracts of the ARAS can be observed resembling a “y”. MFB = Middle Forebrain Bundle, DR = Dorsal Raphè, ARAS = Ascending Reticular Activating System, ROI = Region of Interest.

A manual tracing of each region of interest (ROI) and the corresponding deterministic fiber tracking were performed with FuncTool 9.4.04b™ (© General Electric Medical Systems, MA, USA). Correction of EPI distortions (scaling + translation + shearing) was applied. The ARAS fiber tracts were reconstructed by selection of the fibers passing though the ROIs. The ARAS pathways are so-called neurotransmitter-specific, including fibers coming from specific nuclei. These nuclei include serotonergic in the raphe of the rostral pons and midbrain, noradrenergic in the rostral pons, dopaminergic in the ventral tegmentum of the caudal midbrain, cholinergic in the caudal midbrain and rostral pons, and glutamatergic in the rostral pons. These fibers project to the hypothalamus (regulation of autonomic function and sleep-awake cycles), the thalamus (integration and modulation of arousal brainstem stimuli), the basal forebrain (cortical activation and autonomic integration), and the cortex [1]. In this study, we traced three different ROIs, trying to include all proximal and distal ARAS fiber tracts: the first ROI in the caudal ventromedial mesencephalon, the second in the ventral/anterior thalamus (bilaterally), and the third in the hypothalamus (bilaterally). The hypothalamic ROI was traced in a formatted coronal tensor, in both lateral walls of the third ventricle. Mean, maximal, and minimal fractional anisotropy values were acquired. Each ROI area was also recorded. Each midbrain, thalamic, and hypothalamic ROI served as a seeding point forming a group of fibers with a shape that resembles a “y”, coming from the lower brainstem, ascending through the thalamus, the hypothalamus and finally projecting the distal fibers to the frontal basal lobe and the frontal paracentral cortex bilaterally. Five different tracts were differentiated in the reconstructions: an intra-thalamic gating pathway complex, the dorsal raphe pathway, the medial forebrain bundle, the ventral tegmental tract, and the dorsal tegmental tract (Figure 1).

Case 1

**Figure 2:**
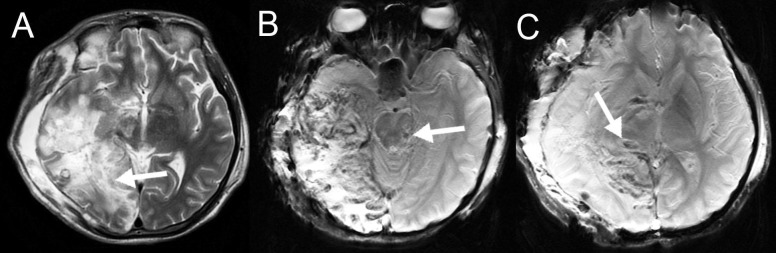
Case 1. Non-enhanced brain magnetic resonance imaging (MRI) after an emergent decompressive craniectomy. (A) Multiple ischemic changes in the right temporal lobe and in the right basal ganglia  areobserved (arrow). (B, C) Susceptibility-Weighted Magnetic Resonance Imaging show hypo-intense images in the mesencephalon and in the right anterior thalamus (arrows), demonstrating grade III diffuse axonal injury.

**Figure 3:**
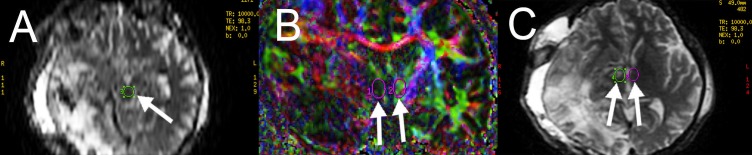
Case 1. Regions of interest (ROIs) traced for multi-seeding in color and T2 fractional anisotropy maps for the ascending reticular activating system (ARAS) fiber tracking. ROIs in the (A) ventromedial mesencephalon, (B) the hypothalamus bilaterally, and (C) the anterior thalamus bilaterally are demonstrated (arrows).

**Figure 4:**
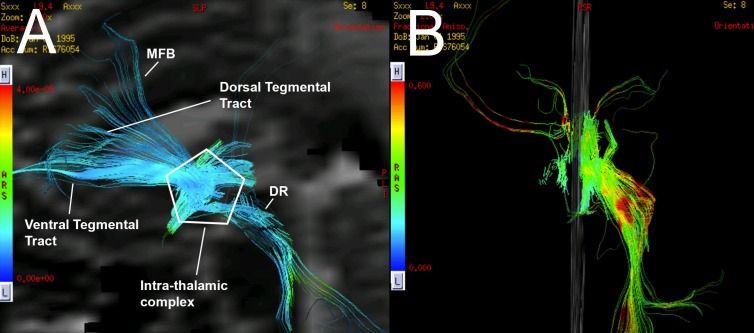
Case 1. Average diffusion coefficient and fractional anisotropy fiber tracking of the ascending reticular activating system (ARAS). Reconstruction of deterministic tractography is observed. (A) A lateral view of the tractography demonstrates the left ARAS fiber tracts. A considerable decrease in the number of the fibers of the DR tract is noted. A partial disconnection between the intra-thalamic complex and the DR tract is also noted. (B) A destruction of the right fibers of the ARAS is observed, including a destruction of the ventral tegmental tract, the dorsal tegmental tract and of the MFB tract. MFB = Middle Forebrain Bundle, DR = Dorsal Raphè, ARAS = ascending reticular activating system.

The patient was a 21-year-old Hispanic male who was admitted to the emergency department six hours after a severe acute TBI caused by a direct trauma while playing soccer. The patient started with headaches and six hours after the trauma started with left hemiparesis and loss of consciousness. He had no medical or familiar remarkable antecedent. Computed tomography (CT) showed a right temporal epidural hematoma with right hemispheric brain edema. The patient underwent an emergent draining of the hematoma with a right decompressive craniectomy. The procedure presented no complication. The patient remained hemodynamically unstable in the ICU for two days. Three days after surgery the sedative medications were withdrawn. Any infection or hydro-electrolytic disorder was ruled out. He was diagnosed with a VS. A non-enhanced brain MRI with the DTI protocol was acquired on day 12 after trauma to clarify the cause of the VS. The images showed an infarction of the right posterior cerebral artery, with an ipsilateral frontal-parietal-temporal transcranial herniation, with multiple subcortical contusions denoted in the susceptibility weighted imaging (SWI), demonstrating a diffuse axonal injury (DAI) grade III (midbrain compromise) (Figure 2). DTI and tractography reconstruction demonstrated destruction of the right fibers of the ARAS (Figures 3-4). The patient remained in VS, then he presented a respiratory sepsis and 60 days after admission the patient deceased.

Case 2

**Figure 5:**
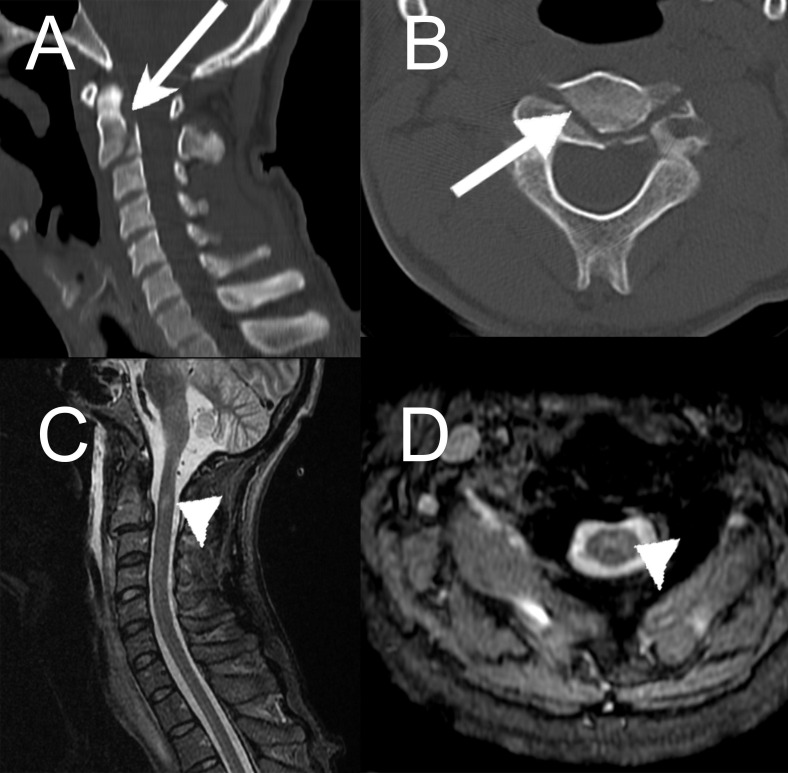
Case 2. Cervical spine computed tomography (CT) and magnetic resonance imaging (MRI) scans. (A, B) A C2-C3 complex fracture (Anderson 3) with traumatic spondylolisthesis (Hangman 2) is observed (arrows). (C, D) A traumatic spondylolisthesis C2-C3 with a C2 contusion with marked myelopathy is demonstrated (arrowheads).

**Figure 6:**
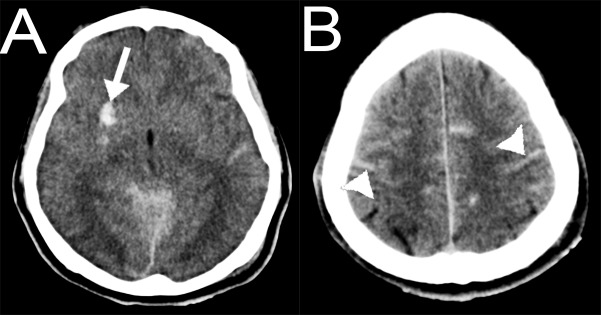
Case 2. Axial computed tomography (CT) of the head. (A) Right thalamic contusions (arrow) with (B) traumatic subarachnoid hemorrhage (arrowheads) are demonstrated.

**Figure 7:**
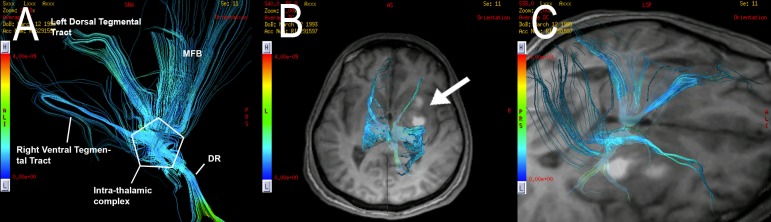
Case 2. Average diffusion coefficient tractography tracing of the ascending reticular activating system (ARAS). (A) A left posterolateral view of the ARAS tractography is presented, demonstrating the ARAS fiber tracts. (B, C) Superior views of a fusion of the tractography and the volumetric axial T1 shows a destruction of the right dorsal tegmental tract in direct relationship with the presence of a right thalamic contusion (arrow). ARAS = Ascending Reticular Activating System, MFB = Middle Forebrain Bundle, DR = Dorsal Raphè.

This was a 32-year-old Hispanic male who was hit by a car when he was walking across a street. After the initial trauma, the patient was found with impaired consciousness and was intubated in the street due to an initial GCS score of three. At the emergency department, he was found under mechanical ventilation, under sedative medication, with a Richmond Agitation Sedation Scale (RASS) score of -5. Pupils were found 4 mm left, 3 mm right, and with brainstem reflexes present. The patient was evaluated for multiple trauma in the thorax and in the abdomen. CT scans of the head and of the spine were performed showing diffuse traumatic subarachnoid hemorrhage in both convexities and DAI grade III, with a C2-C3 complex (Hangman 2/Anderson 3) fracture with anterior luxation (Figures 5-6). An external ventricular drain was inserted. The intracranial pressure remained under 15 mmHG for 72 hours and then it was removed. The C2-C3 spondylolisthesis was treated conservatively with immobilization at first due to hemodynamic instability. The patient remained in a VS after the sedation was discontinued. A spastic quadriparesis was noted as well. A brain MRI with tractography was performed 15 days after the trauma, showing multiple contusions, denoting DAI grade III (right thalamic contusion as well as with a midbrain contusion), with complete destruction of the right dorsal tegmental tract (Figure 7). Cervical spine MRI also showed a C2 spinal contusion with marked associated myelopathy. The patient remained in a VS, during his hospital stay. No neurological change was noted. Finally, the patient presented a cardiorespiratory arrest and deceased 60 days after his admission.

Case 3

**Figure 8:**
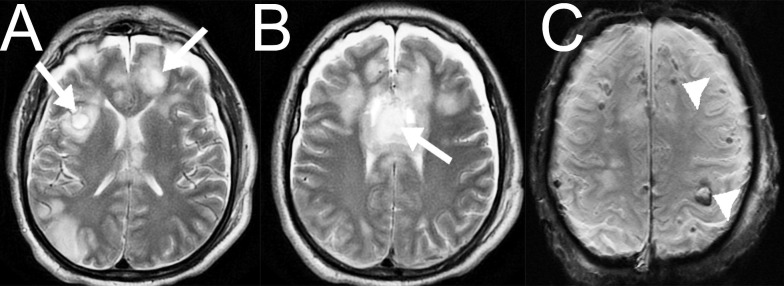
Case 3. Non-contrast magnetic resonance imaging (MRI) of the head. (A, B) Axial T2 weighted imaging (T2WI) and (C) susceptibility-weighted magnetic resonance imaging (SWI) axial slices showing multiple hemorrhagic contusions (arrows) in both frontal lobes as well as in the genu and anterior body of the corpus callosum with corresponding perilesional edema, as well as multiple cortico-subcortical hemorrhagic contusions (arrowheads).

**Figure 9:**
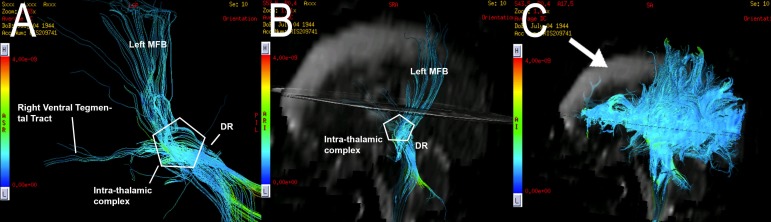
Case 3. Average diffusion coefficient tractography of the ascending reticular activating system (ARAS). (A, B) An anterior and inferior view of the ARAS tractography is presented, demonstrating the ARAS fiber tracts. A destruction of the right dorsal tegmental and right MFB tracts is denoted, as well as a destruction of the left dorsal and ventral tegmental tracts. (C) A destruction of bi-frontal commissural, association, and projection fibers is demonstrated. ARAS = Ascending Reticular Activating System, MFB = Middle Forebrain Bundle, DR = Dorsal Raphè.

This was a 67-year-old Hispanic man who was admitted to the emergency department with a severe acute head trauma after a motor vehicle accident. The patient rated 3T in the GCS. He did not open his eyes, he had no verbal output, and he was without any neurological response. The patient was intubated and remained in a VS during his 32-day hospital stay. A non-contrast MRI of the head was performed showing multiple hemorrhagic contusions, consisting with DAI grade II (corpus callosum compromise) (Figure 8). DTT showed destruction of ipsilateral and contralateral fibers. On the right side, the dorsal tegmental and the middle forebrain bundle (MFB) tracts were destroyed. On the left side, both the dorsal and ventral tegmental tracts were destroyed (Figure 9). At the 12-month follow-up the patient persisted in an MCS.

## Discussion

There are recent studies regarding the reconstruction of the tractography of the ARAS [1-4]. These studies elucidate the neuro-anatomic changes after TBI, defining the potential of recovery of consciousness [3], as well as using tractography as a tool for predicting outcomes or treating DOCs [5]. Moreover, the conventional deterministic tractography is a relatively quick study, though it is costly, and involves the knowledge of the technique for carrying out the examination and the appropriate software and personnel for the post-processing of the acquisitions. For now, it is possible to identify the ARAS with deterministic tractography as demonstrated in this study. New models of probabilistic tractography can even visualize other mathematically possible structural connections of the ARAS [2, 4], giving more information than just FA reconstructed fibers. In both cases, however, the limitation of ellipsoid-based imaging, and little sensitivity for low FA values of the ARAS, a pathway that is both functional than structural, makes it difficult to isolate the arousal system in fiber tracking. Low FA values have been observed previously on the midbrain [9], so this could explain why most of these non-ARAS fiber tracts were not reconstructed in our study. Future segmentation of brainstem nuclei with the proposed atlas of the ARAS [1] will make it easy to reproduce the ROI seeding to compare adequately FA values in isolated ARAS fiber tracts. However, this process is delicate and difficult to perform with 1.5 T imaging information. Indeed, this tool remains operator-dependent and observer-dependent. Further, this instrument needs to be powered to use it as an accurate clinical tool. 

This study provides a detailed description of ARAS fibers, and the quality of the images provided are remarkable. We found that the FA decreases in places where there is the destruction of the ARAS due to DAI. In cases 1, 2, and 3, there was a destruction of ipsilateral fibers to the traumatic lesions; however, in case 3 the patient also presented a contralateral destruction of ARAS fibers. We attempted to correlate the imaging findings with the neurological status of the patient, however, this information is not enough to determine which exact part of the ARAS fiber tracts must be injured to produce any DOC. During the acute and subacute phases of the trauma, all patients presented a VS. Two of them unfortunately deceased, and a correlation for prediction of outcome is not possible. In case 3, the patient presented an important destruction of the fibers, and in the follow-up, a minimal recovery was documented.

Regarding the mean FA values, case 1 presented a decrease mean FA value in the right hypothalamus, case 2 presented decreased FA values in the thalamus bilaterally and in the left hypothalamus, and case 3 presented decreased FA values in the thalamus bilaterally (Tables 1-3). These values were compared to mean FA values of the ARAS tracts in healthy subjects [2]. The abnormal clinical and radiological findings of the patients are presented (Table 4).

**Table 1:** Case 1. FA values of the ROIs traced for fiber tracking. ROI = Region of Interest, FA = Fractional Anisotropy, SD = Standard Deviation.

ROI	ROI area (mm2)	Mean FA	Min. FA	Max. FA	SD
Ventral and medialm esencephalon (superior)	78	0.352	0.186	0.553	0.0899
Left anterior thalamus	47	0.797	0.467	0.9	0.18
Right anterior thalamus	44	0.36522	0.273	0.465	0.047817
Left hypothalamus (coronal tensor)	83	0.41	0.213	0.81	0.106
Right hypothalamus (coronal tensor)	95	0.165	0.062	0.39	0.068

**Table 2:** Case 2. FA values of the ROIs traced for fiber tracking. ROI = Region of Interest, FA = Fractional Anisotropy, SD = Standard Deviation.

ROI	ROI area (mm2)	Mean FA	Min. FA	Max. FA	SD
Ventral and medial mesencephalon (superior)	72	0.352	0.186	0.553	0.0899
Left anterior thalamus	52	0.308	0.208	0.462	0.102
Right anterior thalamus	41	0.297	0.129	0.514	0.097
Left hypothalamus (coronal tensor)	80	0.254	0.058	0.574	0.097
Right hypothalamus (coronal tensor)	66	0.337	0.107	0.484	0.091

**Table 3:** Case 3. FA values of the ROIs traced for fiber tracking. ROI = Region of Interest, FA = Fractional Anisotropy, SD = Standard Deviation.

ROI	ROI area (mm2)	Mean FA	Min. FA	Max. FA	SD
Ventral and medial mesencephalon (superior)	73	0.387	0.150	0.742	0.124
Left anterior thalamus	49	0.322	0.156	0.500	0.092
Right anterior thalamus	49	0.329	0.081	0.838	0.131
Left hypothalamus (coronal tensor)	73	0.441	0.226	0.777	0.149
Right hypothalamus (coronal tensor)	95	0.441	0.065	0.776	0.108

**Table 4:** Remarkable clinical and radiological findings. *The patient changed to a minimally conscious state in the 12-month follow-up. MFB = Middle Forebrain Bundle, ROI = Region of Interes, FA = Fractional anisotropy.

	Case 1	Case 2	Case 3
Initial radiological findings	Edema Right epidural hematoma	Bilateral thalamic contusions Diffuse axonal injury Traumatic subarachnoid hemorrhage	Bi-frontal contusions Diffuse axonal injury
Disorder of consciousness	Vegetative state	Vegetative state	Vegetative state*
Compromised tracts	Right ventral tegmental tract Right dorsal tegmental tract Right MFB	Right dorsal tegmental tract	Bilateral ventral and dorsal tegmental tracts Right MFB
ROIs with decreased FA values	Right hypothalamus	Thalamus bilaterally Left hypothalamus	Thalamus bilaterally

The data in the cases of this paper show some agreement to previous works that describe cases about injury of the ARAS [2-4]. It is not yet clear if a unilateral damage in the brainstem or other parts of the ARAS is in direct relationship with consciousness impairing. However, a cohort study demonstrated that central lesions or unilateral brainstem lesions on early MRI were modestly associated with a worse outcome in severe TBI [10]. A description of ARAS fibers reconstructed with 1.5 T scanners has been reported elsewhere [2, 4]. However, to our knowledge, this is the first study with a 1.5 T magnet, where the ARAS fibers are described in detail, denoting the ventral tegmental tract, the dorsal tegmental tract, the intra-thalamic complex, the dorsal raphè (DR), and the MFB tracts. Reconstructing the ARAS in this way allows physicians to do it in a short period (6-7 minutes) in a clinical field, making it more feasible to use rather than other techniques like high angular resolution diffusion imaging (HARDI) tractography, where one scan can take up to five hours to be performed [1]. Another limitation of this study was to carry out a sub-millimetric analysis of the fibers; differentiation of lateral and middle fibers of the dorsal tegmental tract and the ventral tegmental tract was not possible in this work. Further studies should be performed with normal subjects; evaluating the mean diffusivity and the number of fibers in each subject, thus determining the variability of these parameters within an expected normal range.

We found that there was an asymmetry in the number of fibers reconstructed including between both sides of each subject. It is also necessary to establish the appropriate size ranges for multi-seeding because connections may vary between subjects. In patients with TBI and a DOC, it is known that there is a destruction of tracts and there is a disruption of the pathway, but not necessarily bilaterally. It is possible to reconstruct the ARAS with multiple two-dimensional ROIs located in the ventromedial mesencephalon, the anterior thalamus bilaterally, and in the hypothalamus bilaterally (including the most important nuclei: the ventral tegmental area, the dorsal raphé, the peduncule-pontine nucleus, the central lateral nucleus of the thalamus, and the centromedian/parafascicular complex of the thalamus) [1].

With the advent of multidisciplinary approaches for the treatment of TBI, mortality in these patients has decreased. Patients survive severe TBI with higher rates, remaining in any DOC [3]. The economic and social impact in this regard is invaluable. Despite giant efforts to elucidate the problem, the basis of TBI and the degree and pattern of ARAS injury remains unclear [3]. We find it very valuable that tractography of the ARAS could help in prediction of the prognosis of patients who are at an intermediate point between different DOCs after TBI. Additional information on functional magnetic resonance imaging (fMRI) and new techniques with resting-state fMRI will proportionate integrated information about the connectivity of the ARAS.

## Conclusions

The tractography shows the reconstruction of the ARAS, demonstrating few fibers ascending through the brainstem. The ventral tegmental tract, the dorsal tegmental tract, the DR, the MFB, and the intra-thalamic complex tracts fibers were reconstructed. Affected fibers of the ARAS with DAI and the FA values abnormalities in the ARAS in three patients with DOC after TBI are described. Future automated seeding based on the FA values will reduce the inter-observer/inter-operator differences for this reconstruction. Additional studies with longer follow-ups will address more information regarding prognosis in these patients.
